# Profile differences between overdose and non-overdose suicide attempts in a multi-ethnic Asian society

**DOI:** 10.1186/s12888-016-1105-1

**Published:** 2016-11-08

**Authors:** Cyrus S. H. Ho, Y. L. Ong, Gabriel H. J. Tan, S. N. Yeo, Roger C. M. Ho

**Affiliations:** 1 Department of Psychological Medicine, Yong Loo Lin School of Medicine, National University of Singapore, Level 9, NUHS Tower Block, 1E Lower Kent Ridge Road, Singapore, 119 228 Singapore; 2 Duke-NUS Graduate Medical School, Singapore, Singapore

**Keywords:** Overdose, Non-overdose, Suicide attempts, Multi-ethnic, Asian

## Abstract

**Background:**

This study explores differences in characteristics of overdose (OD) and non-overdose (NOD) suicide attempts in Singapore.

**Methods:**

Four hundred eighty-five medical records of people who attempted suicide were extracted from a local general hospital patient database and classified into OD and NOD groups. Differences in socio-demographic factors, suicide characteristics and hospital admission types between both groups were examined.

**Results:**

Indians were more likely than the Chinese and Malays to employ OD method in their attempts. More suicide attempts in the OD group than NOD group were self-reported. The most likely place for suicide attempts for both groups was at home, though more NOD suicide attempts were in public areas as compared to the OD group. Analgesics were the most used substance in the OD group. Those who attempted suicide using OD had a higher number of psychiatric ward admissions than the NOD group. Risk and protective factors varied between both groups.

**Conclusion:**

Differences in socio-demographics, suicide characteristics and admission characteristics between OD and NOD groups were observed. Recommendations for suicide prevention in the community are discussed. Further studies on the mediators and moderators of these trends and characteristics of suicide attempts are necessary to ensure maximal efficacy of prevention and management.

## Background

Suicidal behaviour is a global public health concern that leads to not only an immense financial burden on society, but also incurs a hefty psychological toll on survivors and their loved ones. Each year, 10–20 million individuals attempt suicide and over 800,000 lives are lost [1]. Despite the fact that Asian countries account for about 60 % of global suicides [2], there is inadequate resources to tackle the magnitude of the problem in this region. This is compounded by the lack of reliable statistics and rigourous suicide research. While extant literature has data on epidemiologic characteristics and trends of suicide methods common in Asia [3], the gaps in knowledge on the influence of various characteristics and risk factors on different suicide methods persist. Suicide methods have been found to be particularly influenced by the availability, knowledge, past experiences, symbolism, cultural significance, and mental state of the attempters [4]. The psychosocial needs of suicidal individuals could also contribute to the chosen method of suicide [5]. Differences in methods may lead to different outcomes such as repetitive suicide attempts or eventual suicide completion [6, 7], which have differing implications on the regional health system and financial costs. Modification of the environment by restricting access to lethal means such as firearms, pesticides and charcoal burning have been found to reduce method-specific suicide rates, and when the method is prevalent, it could lead to decline in the overall suicide rate [8]. Nevertheless, there have also been other studies that suggested restriction of certain means might not effectively reduce suicide risk as it might prompt suicidal individuals to consider alternative means of suicide, which potentially could be more fatal [9]. Therefore, it is imperative that a greater emphasis be placed on understanding the intricacies of suicidal behaviour in Asia by elucidating the suicide characteristics of each country and culture. The World Health Organisation (WHO) recommends the enhancement of suicide prevention efforts by improving the availability and quality of data from hospital-based systems [10]. The knowledge acquired from such data will play a pivotal role in the formulation and implementation of targeted suicide prevention measures.

Singapore which is ranked 97^th^ in the world in terms of number of suicide deaths [11], is a culturally diverse society (Chinese: 74.3 %, Malay: 13.3 %, Indian: 9.1 %, Others: 3.2 %) in Southeast Asia [12]. The most common methods of suicide in Singapore include jumping from height, hanging, and poisoning [13]. Most studies of suicide in Singapore are based on data obtained from the Registry of Birth and Death, and from Coroner’s Court reports [14, 15] and few studies have analysed the data from hospitals. As part of standard operating procedures in the hospital systems of Singapore, a patient admitted following his or her suicide attempt is required to undergo psychiatric assessment. Data from such assessment holds valuable information such as socio-demographics and characteristics of suicide attempters that can guide future policies and protocols in suicide prevention. The study was therefore conducted to compare and contrast the profiles of suicide attempters. As jumping from height and hanging are more likely than poisoning to lead to suicide completion and thereby resulting in the lack of data of interest, we classified the attempters into overdose (OD) and non-overdose (NOD) groups for comparison. In this study, people who took medication at higher doses beyond what was recommended by doctors with an intention to commit suicide were labeled under the OD group. As there have been few studies comparing the suicide profile of people who overdosed on drugs compared to those who used other methods, this would be a pertinent area of study. With variation in the nature, circumstances and lethality of suicide method between OD and NOD groups, we hypothesised that there would be differences in socio-demographics, suicide characteristics, as well as risk and protective factors between the two groups. The present study also serves as an update to a previous study done between 2004 to 2006 [16] and explores the differences in characteristics between OD and NOD attempters in Singapore.

## Methods

### Study design

A total of 485 assessment records of admitted adult suicide attempters aged 21 years old and above from January 2009 to December 2012 were extracted from a general hospital (National University Hospital, Singapore) database. Data sources included the suicide risk assessment forms, psychiatric reports, in-patient records and electronic notes. The assessment was done by the psychiatrist and this was part of the protocol of standard operating procedures for patients admitted following a suicide attempt. The interview was based on a semi-structured checklist approach. A formal diagnosis was made by the psychiatrist based on the Diagnostic and Statistical Manual of Mental Disorders Fourth Edition (Text Revision) (DSM-IV-TR) criteria.

The suicide risk assessment form that was used in the study contained stressors that comprised of work-related issues, family-related issues, relationship problems, financial difficulties and medical illnesses; risk factors that included history of psychiatric illnesses, family history of psychiatric illnesses, staying alone, alcohol or substance misuse, ongoing interpersonal problems, lack of confidantes, serious physical illnesses, poor coping skills, severe financial problems and unemployment; and protective factors that consisted of faith in a religion, resolution of precipitants, receiving support from dependents, expression of regret, positive plan for the future, willingness to seek help and good emotional support. All suicide attempters were also asked to rate their impression of lethality of their suicide method as part of the suicide risk assessment, which ranged from ‘not lethal’, ‘moderately lethal’ to ‘very lethal’.

This assessment form was created by a team of psychiatrists in the department based on known common risk factors of suicide. The initial and main aim of the checklist form was to serve as a clinical tool for the assessing psychiatrist to avoid missing out on the common possible stressors and risk/protective factors. To standardize the assessment, all the psychiatrists were briefed and trained on asking the questions in the form so that they could reach a common consensus on the terms and what they meant prior to using the form.

Attempters were classified into either the OD group if there were indications of consumption of pharmacological or chemical substances in the attempt or the NOD group for all other forms of deliberate self-harm, including lacerations, stabbing, jumping from height and hanging. Written informed consent was obtained from all the participants in the study. The study was approved by the Domain Specific Review Board (DSRB) (DSRB Reference: 2013/00800).

### Statistical analysis

The Statistical Packages for Social Sciences (SPSS) Version 21.0 software was used to perform statistical analysis. The Chi-square test was conducted to examine differences in characteristics and suicidal behaviours between and within the OD and NOD groups. The Mann-Whitney U test was employed to determine differences in hospital admission data between the OD and NOD attempters. The assumption of similarly shaped distributions was ascertained by visual inspection. Logistic Regression was used to test for differences between the self-perceived lethality of the suicide methods employed between OD and NOD groups.

## Results

### Socio-demographic and characteristics of attempts

In both the OD and NOD groups (Table 1), most of the attempters were above the age of 21. Majority of the attempts were also unplanned, precipitated by relationship, family, or work problems, and took place on weekdays. Acute stress reaction, depressive disorder and adjustment disorder were the top three psychiatric diagnoses observed in both groups.

**Table 1:** Comparison of the characteristics of suicide attempters from 2009 to 2012 (*N* = 485)

	Overdose		Non-overdose			
	N	%	N	%	χ^2^	*P*
Gender						
Male	150	(35.8)	31	(47)	3.29	0.07
Female	269	(64.2)	35	(53)		
Age						
Below 21	56	(13.4)	9	(13.6)	1.75	0.78
21–30	114	(27.2)	18	(27.3)		
31–40	88	(21)	16	(24.2)		
41–50	77	(18.4)	14	(21.2)		
50+	84	(20)	9	(13.6)		
Ethnicity						
Chinese	265	(63.4)	38	(56.7)	16.312	0.01*
Indian	80	(19.1)	9	(13.4)		
Malay	39	(9.3)	5	(7.5)		
Indonesian	8	(1.9)	6	(9)		
Others	26	(6.2)	9	(13.4)		
Discovery of Suicide attempt						
Self-Reported	133	(31.8)	7	(10.4)	34.037	<0.01**
Father or Mother	37	(8.9)	7	(10.4)		
Son or Daughter	29	(6.9)	3	(4.5)		
Spouse	52	(12.4)	3	(4.5)		
Sibling	21	(5)	3	(4.5)		
Friend	36	(8.6)	8	(11.9)		
Boyfriend or Girlfriend	10	(2.4)	4	(6)		
Other relatives	16	(3.8)	1	(1.5)		
Others	84	(20.1)	31	(46.3)		
Location of Suicide Attempt						
Home	340	(81.1)	40	(60.6)	20.356	<0.01**
Home of other people (e.g. friend)	7	(1.7)	0	(0)		
Workplace	10	(2.4)	3	(4.5)		
Public areas or other areas	62	(14.8)	23	(34.8)		
Suicide attempt took place during weekend						
Yes	103	(24.6)	14	(21.2)	0.354	0.55
No	316	(75.4)	52	(78.8)		
Planning before Suicide Attempt						
Yes	52	(12.4)	8	(12.1)	0.000	1.00
No	367	(87.6)	58	(87.9)		
Admitting Suicide Intention						
Yes	208	(49.6)	35	(53)	0.058	0.809
No	211	(50.4)	31	(47)		
Precipitants						
Work-related issues	122	(29.1)	27	(40.9)	2.232	0.14
Family-related issues	164	(39.1)	29	(43.9)	0.691	0.41
Relationship problems	212	(50.6)	34	(51.5)	0.017	0.90
Financial problems	98	(23.4)	17	(25.8)	0.002	0.97
Medical Illnesses	68	(16.2)	11	(16.7)	0.000	1.00
Other Issues	30	(7.2)	6	(9.1)	0.092	0.76
Psychiatric Diagnosis						
Acute Stress Reaction	171	(40.8)	26	(39.4)	0.016	0.90
Adjustment Disorder	61	(14.6)	10	(15.2)	0.000	1.00
Depressive Disorder	76	(18.1)	17	(25.8)	1.165	0.28
Bipolar Disorder	2	(0.5)	0	(0)	0.000	1.00
Schizophrenia	6	(1.4)	1	(1.5)	0.000	1.00
Borderline Personality disorder	10	(2.4)	1	(1.5)	0.003	0.96
Alcohol abuse/dependence	1	(0.2)	1	(1.5)	0.191	0.66
Misuse of other substances	0	(0)	1	(1.5)	1.051	0.31
Other diagnosis	92	(22)	9	(13.6)	2.430	0.12

(*P < 0.05; **P < 0.01)

In comparing demographics and characteristics of attempts between the OD and NOD groups (Table 1), ethnicity, discovery of suicide attempt, and location of suicide attempt were significantly different. After adjustment for population size of each race, Indians had the highest number of suicide attempts at 22.8 cases per 100,000 people, followed by Chinese and Malays at 9.36 and 7.65 cases per 100,000 people respectively. Trends were similar for the NOD group, with Indians, Chinese and Malays at 2.56, 1.34, 0.98 cases per 100,000 persons respectively. Suicide attempts by the OD group were also more likely to be self-reported as compared to attempts by the NOD group which were more likely to be discovered through other reporting methods. Furthermore, while the most likely place for both OD and NOD suicide attempts was at home, significantly more NOD than OD suicide attempts were in public areas. Finally, the difference between frequencies of males and females in the OD and NOD groups neared significance, tending to suggest that more males than females attempted suicide using NOD methods (Table 1). Patients in the OD group also had significantly more psychiatric admissions than NOD attempters in the period between 2009 and 2012 (Table 2).

**Table 2:** Mean and Median frequency of Emergency Department (ED) consultations, psychiatric ward (Psy) admissions and medical ward (Med) admissions per patient

		ED	Psy**	Med
Non OD	Median	2.00	1.00	1.00
	Mean	4.70	1.09	2.39
OD	Median	3.00	1.00	1.00
	Mean	5.48	2.01	3.49

(**P < 0.01)

### Risk and protective factors

Significant risk factors for attempters in the OD group were living alone, alcohol and substance abuse, and lack of confidantes, while a significant protective factor was to have resolution of the precipitating factors. On the other hand, a significant protective factor for attempters in the NOD group was the willingness to seek help and having good emotional support (Table 3).

**Table 3:** Comparisons of risk and protective factors for suicide attempts between non-overdose and overdose cases (*N* = 485)

Factors	Overdose	Non-overdose	χ^2^	*P*
Risk Factors				
History of psychiatric illnesses	62.0	66.6	0.57	0.45
Family history of psychiatric illnesses	85.5	86.1	0.01	0.91
Living alone	92.9	80.5	11.70	<0.01**
Alcohol or substance misuse	82.8	70.8	5.82	0.02*
Ongoing interpersonal problems	41.8	36.1	0.85	0.36
Lack of confidantes	68.8	51.3	8.48	<0.01**
Serious physical illnesses	85.8	80.5	1.35	0.25
Poor coping skills	67.9	69.4	0.06	0.80
Severe financial problems	81.0	77.7	0.41	0.52
Unemployment	77.1	68.0	2.77	0.10
Protective Factors				
Faith in a religion	44.3	51.3	1.22	0.27
Resolution of precipitants	80.7	68.0	6.03	0.01*
Receiving support from dependents	49.4	55.5	0.93	0.34
Expression of regret	28.8	20.8	1.98	0.16
Positive plan for the future	34.0	37.5	0.32	0.57
Willingness to seek help	13.9	25.0	5.75	0.02*
Good emotional support	24.7	38.8	6.34	0.01*

(*P < 0.05; **P < 0.01)

### Characteristics of attempts in OD group

Of special interest was the type of drugs used by the attempters in OD group. While a wide of range of drugs was used, the five most common drugs of choice were analgesics, hypnotic agents, domestic products, antidepressants, and flu medication. Most attempters consumed only one drug (Table 4).

**Table 4:** Drugs consumed in OD group (*N* = 419)

	Number	Percent
Analgesics	183	(43.7)
Hypnotic agents	125	(29.8)
Domestic products (e.g. detergents and cleaning solutions)	56	(13.4)
Diabetes drugs	10	(2.4)
Flu medications or antibiotics or antihistamines	43	(10.3)
Antidepressants	48	(11.5)
Medications for gastrointestinal systems (e.g. antacids, antiemetic)	12	(2.9)
Supplements	1	(0.2)
Antihypertensive	12	(2.9)
Opioid	19	(4.5)
Antipsychotics	29	(6.9)
Other drugs or substances	59	(14.1)
Patient consumed only one drug or substance	277	(66.1)
Patient consumed two or more drugs or substances	142	(33.9)

### Self-perceived lethality of the suicide methods

Only 46.7 % of female OD suicide attempters perceived their attempts as lethal as compared to 58.6 % of males perceiving their attempts as lethal (OR = 0.62, *p* = 0.02). Chinese OD suicide attempters had higher perceived lethality of their suicide methods (OR = 0.64, *p* = 0.03) as compared to all other ethnicities. All other demographic factors did not have significant impact on their perceived lethality (Table 1).

### Frequency and distribution of suicide attempts across Singapore

The lowest number of suicide attempts occurred on Fridays (46 attempts, 9.48 %) and Saturdays (47 attempts, 9.69 %) for both OD and NOD groups, whilst the highest occurred during mid-week on Wednesdays for the OD group (75 attempts, 15.46 %) and on Thursdays for the NOD group (13 attempts, 2.68 %). The lowest number of attempts was in the month of January for both OD and NOD groups (16 attempts, 3.30 %), whilst the highest occurred in the month of May for the OD group (50 attempts, 10.31 %) and in April for the NOD group (12 attempts, 2.47 %).

An exploratory analysis of geographical distribution of attempts showed that areas in Singapore with more than 20 suicide attempts had a high density of high rise residential buildings. Presence of industrial estates that is typically more isolated had little impact on the number of suicide attempts.

## Discussion

### Socio-demographic factors

Our results suggested that ethnicity may influence the choice of suicide method, with Indians tending to choose OD over NOD methods. This finding is also in concordance with several studies of completed suicides carried out in Malaysia [17–19], a neighbouring country of Singapore which also has these three ethnicities (Malay, Chinese, Indian) in their population make-up. Due to lack of data from the sources, it was a challenge to identify the motivating factors for choosing OD over NOD methods. However, the near significant gender difference in suicide methods observed in our results, mirrored evidence in the literature that women tend to use less violent suicide methods like poisoning [20, 21] as compared to men who tend to use NOD methods such as hanging [22].

### Precipitants and risk factors

Majority of the attempts in both groups were unplanned, precipitated by relationship, family, or work problems and some attempters were observed to have acute stress reaction, depressive disorder or adjustment disorder. This is supported by a study by Chia et al. [23] that identified relationships and job- related problems as social risk factors of suicide in Singapore. Non-Chinese OD suicide attempters and females were also more likely to perceive their suicide as less lethal. Individuals who make impulsive attempts tend to carry out their plans without pre-meditation and expect their method to be less lethal than the actual lethality [24]. Therefore, it is possible that some of the suicide attempts might have been carried out on impulse due to inability to cope with life stressors. Further research will be required to delve deeper into the mechanisms behind impulsive NOD and OD suicide attempts.

Attempters who lived alone or had a lack of confidantes had a higher risk of attempting suicide with OD methods than the NOD group. There were also fewer protective factors in the OD group, as attempters did not have good emotional support and were less willing to seek help. Social support has been found to be associated with decreased likelihood of a lifetime suicide attempt [25], suggesting that providing timely social support to attempters in times of need might help to reduce the number of OD attempts.

### Characteristics of OD attempts

The most common drugs consumed in the OD group (analgesics, hypnotic agents, domestic products, flu medications and antidepressants) can be easily purchased over the counter or obtained through prescription by a general practitioner for sleep and mood disorders. Evidently, the ease of obtaining a substance for OD is of major importance in suicide attempts. Means restriction is an important population strategy for suicide prevention [26]. Thus, if high enough barriers to lethal drugs are imposed, it may lead to a reduction in likelihood of overdosing on a particular substance [27] and reconsideration of the impulse to attempt suicide [28]. Further research is recommended to ascertain the need for more rational prescribing and stricter control over quantities of common medications that can be bought over the counter.

### Time and geographical patterns of suicide attempts

Different periods of a week or year may lead to variation in the number of suicide attempts due to differing environmental conditions and stressors [29]. Fridays leading up to weekends correspond to times of less stress and therefore leading to a fall in number of attempts in our study. Number of suicide attempts increased again just before the start of the week on Sundays. There is a build-up of stress levels on weekdays from Mondays to Thursdays, leading to increased suicide attempts. This is also consistent with the trends for completed suicides [30, 31]. Since the months of March, April and May are the hottest months in Singapore [32], higher ambient temperatures leading to increased stress in attempters may correspondingly explain the highest number of suicides in these months. Similar reports have been made for completed suicides [33, 34], and this is postulated to have underlying sociological, biological, or psychological mechanisms which are still under intensive research [35, 36]. The lowest number of suicide attempts in January and February can be explained by the fact that they are the first few months of the year with major national holidays which could be associated with the positive outlook that things will change for the better. This is in accordance with major holidays being preceded by lower number of suicide attempts [37, 38].

Additionally, our data on geographical distribution indicated that attempts tended to occur at home and in high density residential areas. This provides insight on how suicide prevention or intervention strategies can be formulated to reach out to attempters or people contemplating suicide in their homes.

### Strengths and limitations of study

While our study included a large sample size of patients, it is not without limitations. Firstly, the sample was obtained from only one general hospital and it only included those with attempted but not those who completed suicide. Our sample consisted of mainly younger adults and not the elderly, which reduces generalizability of the findings to other age groups. Furthermore, the NOD group had a small sample size and thus might not have been representative of the NOD suicide attempters of Singapore. Within this group, there was heterogeneity in terms of the method of suicide, which varied from laceration to jumping from height and hanging, and this carried different degrees of lethality. The measures indicated in the suicide risk assessment form were based on clinician judgement and there was no inter-rater reliability for assesment. The cross sectional nature of the data prevented the understanding and prediction of later suicide risk. There was also possibility of recall bias in this archival retrospective study. In addition, other relevant moderators and mediators of OD and NOD methods such as personality characteristics, coping methods, history of suicide ideation or attempts were not documented. Despite these limitations, our study provides valuable information on the dynamic characteristics of suicide attempts in Singapore, which could help guide the development of policies and intervention methods suited to current trends.

## Conclusion

Due to the changes in patient risk factors over time, it is important to keep abreast of the characteristics of current suicide attempts, so as to guide the government in developing up-to-date strategies for the prevention of future suicide attempts [39]. Information generated from our study would be an important addition to the current knowledge pool on suicide and contribute to the subsequent development of new treatment methods [14] and may also validate the efficacy of current treatment methods [40, 41].

In a recent study conducted by Chong et al. [42], majority of Singaporeans (68.3 %) who were suffering from mental disorders failed to seek help, and 55.6 % of individuals thought that they could handle the problem on their own, while 31.6 % were oblivious of their mental condition. This, together with the data from our study, hints at the importance of encouraging individuals to have greater awareness of mental health issues, and increasing the platforms of help available (e.g. suicide or crisis hotlines) through use of new media such as the social media and smartphone applications. Additionally, policymakers and mental healthcare providers should evaluate and improve on current protocols in identifying and supporting psychiatric patients with suicidal ideation. Since repeated psychiatric admission is predictive of an eventual completed suicide via the OD method, standard care in a psychiatric healthcare setting may have to include more timely follow-up sessions and changes in drug prescription for patients who fall into this category. Finally, public education and outreach programs in the community can empower individuals with the relevant knowledge of identification of suicidal behaviours and signs of deliberate self-harm in their peers and loved ones.

In view of the significant number of suicide attempts being committed at home, another potential suicide prevention strategy would be to foster greater social support among the community. Since individuals who live alone are more prone to repetition of acts of deliberate self-harm [43], closer relationships with neighbours will promote social inclusion and reduce the possibility of repeated suicide attempts. In addition, regional health systems can also improve its accessibility to people at risk of suicide, thereby lowering the barriers to self-reporting of suicide attempts. This may include setting up of more social service centres near densely populated areas to increase accessibility to social resources, and ensuring integration of care between these service centres and other healthcare services.

Future research in the direction of teasing out the factors that motivate people of different ethnicities to choose different methods of suicide, and other risk and protective factors that mediate and moderate OD and NOD suicide attempts, will allow for a more complete understanding of suicide in the Asian context.
